# Profiles of Small Regulatory RNAs at Different Growth Phases of *Streptococcus thermophilus* During pH-Controlled Batch Fermentation

**DOI:** 10.3389/fmicb.2021.765144

**Published:** 2021-11-30

**Authors:** Gefei Liu, Haode Chang, Yali Qiao, Kai Huang, Ao Zhang, Yu Zhao, Zhen Feng

**Affiliations:** ^1^Key Laboratory of Dairy Science, Ministry of Education, College of Food Science, Northeast Agricultural University, 600 Chanjiang Road,150030, Harbin, Heilongjiang, China; ^2^Spice and Beverage Research Institute, Chinese Academy of Tropical Agricultural Sciences, Wanning, 571533, Hainan, China; ^3^College of Food and Biological Engineering, Qiqihar University, 42 Wenhua Road, 160006, Qiqihar, China

**Keywords:** *Streptococcus thermophilus*, small RNA sequencing, sRNAs, target genes, expression profiles

## Abstract

Small regulatory RNA (sRNA) has been shown to play an important role under various stress conditions in bacteria, and it plays a vital role in regulating growth, adaptation and survival through posttranscriptional control of gene expression in bacterial cells. *Streptococcus thermophilus* is widely used as a starter culture in the manufacture of fermented dairy products. However, the lack of reliable information on the expression profiles and potential physiological functions of sRNAs in this species hinders our understanding of the importance of sRNAs in *S. thermophilus*. The present study was conducted to assess the expression profiles of sRNAs in *S. thermophilus* and to identify sRNAs that exhibited significant changes. A total of 530 potential sRNAs were identified, including 198 asRNAs, 135 sRNAs from intergenic regions, and 197 sRNAs from untranslated regions (UTRs). Significant changes occurred in the expression of 238, 83, 194, and 139 sRNA genes during the lag, early exponential growth, late exponential growth, and stationary phases, respectively. The expression of 14 of the identified sRNAs was verified by qRT-PCR. Predictions of the target genes of these candidate sRNAs showed that the primary metabolic pathways targeted were involved in carbon metabolism, biosynthesis of amino acids, ABC transporters, the metabolism of amino and nucleotide sugars, purine metabolism, and the phosphotransferase system. The expression of the predicted target genes was further analyzed to better understand the roles of sRNAs during different growth stages. The results suggested that these sRNAs play crucial roles by regulating biological pathways during different growth phases of *S. thermophilus*. According to the results, sRNAs sts141, sts392, sts318, and sts014 are involved in the regulation of osmotic stress. sRNAs sts508, sts087, sts372, sts141, sts375, and sts119 are involved in the regulation of starvation stress. sRNAs sts129, sts226, sts166, sts231, sts204, sts145, and sts236 are involved in arginine synthesis. sRNAs sts033, sts341, sts492, sts140, sts230, sts172, and sts377 are involved in the ADI pathway. The present study provided valuable information for the functional study of sRNAs in *S. thermophilus* and indicated a future research direction for sRNA in *S. thermophilus*. Overall, our results provided new insights for understanding the complex regulatory network of sRNAs in *S. thermophilus*.

## Introduction

Small regulatory RNAs (sRNAs), generally 50–400nt in length, are well established as key genetic regulators in prokaryotes (Wagner and Romby, 2015). sRNAs act as crucial regulators in various processes by exerting posttranscriptional control of gene expression by binding to target genes (Massé and Gottesman, 2002; Jacques et al., 2006). In addition to enhancing or inhibiting mRNA degradation and/or translation by base pairing with mRNA targets, some sRNAs affect the activity of regulatory proteins by chelating regulatory proteins, thereby regulating gene expression (Klein and Raina, 2017). In recent years, there has been great progress in understanding the mechanisms and regulation of bacterial sRNAs. Although sRNA is widely present in prokaryotes, research on the regulation and biological functions of sRNA in the physiology, metabolism and stress response in lactic acid bacteria (LAB) is limited. The full extent of the importance of sRNAs in LAB is unclear, although several studies have indicated that sRNAs play important roles in LAB (Dequan et al., 2015; van der Meulen et al., 2016, 2017; Qi et al., 2017; Miao et al., 2018; Wang et al., 2018; Wu et al., 2018; Andersen et al., 2019; Tian et al., 2019; Gao et al., 2020; Yuki et al., 2020). A 6S RNA involved in carbon source uptake was the first sRNA to be identified in *Lactococcus lactis* MG1363 (van der Meulen et al., 2016, 2017). sRNAs s015, s042, and anti41 were shown to improve nisin yields by increasing the acid tolerance of *L. lactis* F44 (Qi et al., 2017; Miao et al., 2018; Wu et al., 2018). sRNA s015 directly combines with the target genes *pG*, *busAB*, *cysD*, *ilvB*, *tcsR*, *ung*, *yudD*, and *ywdA* in *L. lactis* F44 and improves the expression of these genes. sRNA s042 can directly activate the target genes *argR* and *accD* in *L. lactis* F44. The overexpression of sRNA anti41 can inhibit the expression of *glnR* in *L. lactis* F44. In *Lactobacillus casei-Pediococcus*, sRNA rli28 plays an important role in inhibiting the synthesis of lactic acid, regulating the growth of cells in the logarithmic phase, and maintaining the survival of cells in the stationary phase (Wang et al., 2018; Yuki et al., 2020).

*Streptococcus thermophilus* is an essential LAB that is commonly used in the commercial production of yogurt and cheese. This species is considered to be the second-most important industrial dairy starter after *L. lactis* (Prajapati et al., 2013). During the preparation of starter cultures and the actual fermentation process, fluctuations in temperature, osmolarity, pH, and nutrient availability cause significant stress to *S. thermophilus*. However, although sRNAs have been shown to play important roles in other bacteria, the expression profiles and potential physiological functions of sRNAs have not yet been assessed in *S. thermophilus* (Hoe et al., 2013). Only a few sRNAs have been identified in *S. thermophilus*. The analysis of *S. thermophilus* sRNome shows that many sRNAs are associated to the bacterial immune system known as CRISPR-Cas system (Zorgani et al., 2016). Northern blot profiling revealed the presence of a tracrRNA located upstream of the *cas9* gene of the CRISPR3-Cas in *S. thermophilus* LMD-9 genome, which is involved in crRNA biogenesis through the pre-crRNA maturation (Karvelis et al., 2013). Furthermore, five csRNAs were predicted on *S. thermophilus* CNRZ1066 genome and one csRNA on the plasmid pSt0 which was verified by northern blot (Marx et al., 2010). The current understanding of the expression profiles and potential physiological functions of sRNAs in *S. thermophilus* is insufficient, hindering the ability to study their physiological roles in *S. thermophilus*.

*Streptococcus thermophilus* MN-ZLW-002 is a strain widely used in the production of industrial yogurt. The present study was conducted with this strain as the target strain to assess and analyze the expression profiles of sRNAs. Significant changes in sRNAs were identified. Target genes of sRNAs were predicted and further analyzed for functional categorization among biological pathways. The overall goal of this study was to lay a foundation for future studies on the physiological roles of sRNAs in *S. thermophilus*. To the best of our knowledge, this is the first study to reveal the expression profiles of sRNAs in *S. thermophilus*.

## Materials and Methods

### Strains, Culture Conditions, and Fermentation Experiments

**Figure 1:**
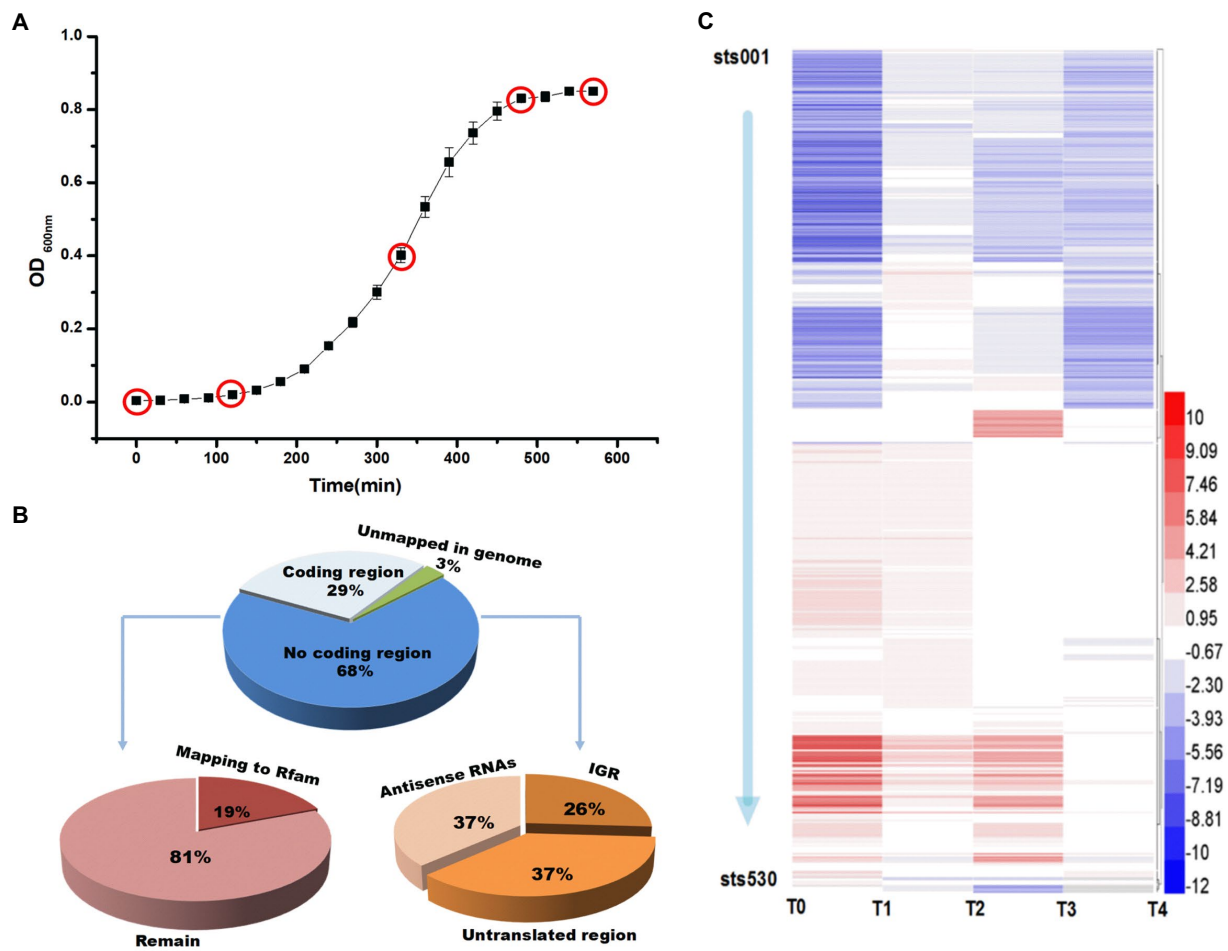
**(A)** Growth curve and five sampling points during culture of ST-MZ-2. The *x* axis represents the culture time. **(B)** Pie charts of the classification of small RNA-sequencing reads. Percentages of all small RNA-sequencing reads that mapped to the genome in annotated or unannotated regions and relative percentages of the reads that mapped to unannotated regions. **(C)** Differential expression of 530 sRNAs. A heatmap showing the 530 sRNA genes identified as being differentially expressed in each growth phase. Blue: upregulated, red: downregulated. The color intensity is a measure of the fold change. T0, T1, T2, T3, and T4 indicate the early-lag, late-lag (early-exponential), mid-exponential, late-exponential, and stationary phases, respectively.

*Streptococcus thermophilus* MN-ZLW-002 was obtained as described in a previous study (Kang et al., 2012). Culture stocks were stored at −80°C. Before use, three subcultivation steps were performed in chemically defined medium (CDM). The CDM was prepared according to the method described by Lahtvee et al. (2011). Batch fermentation was performed in a 10-L Biotech-7,000 bioreactor (Shanghai Baoxing, Shanghai, China) containing 7L of CDM. Starter cultures were centrifuged (10,000×*g*, 10min, 4°C), after which the cells were washed twice with PBS buffer (50mM, pH 6.5) and inoculated in the bioreactor. The temperature and rotation speed were set at 42.5°C and 200 rev/min, respectively. The pH was maintained at 6.25 through the automatic addition of 1M NaOH. Samples were collected during fermentation at five time points (Figure 1A). Each culture sample was centrifuged (12,000×*g*, 4°C, 15min), after which the supernatant was discarded, and the pellet was snap-frozen in liquid nitrogen. Each set of culture conditions was repeated three times.

### RNA Extraction, Library Construction, and Sequencing

Total RNA was isolated using the hot phenol method and the TRK-1002 Kit (LC Bio, China). Next, an Agilent 2,100 Bioanalyzer (Agilent Technologies, Waldbronn, Germany) was used to inspect the integrity of the 16S/23S rRNA and to assess DNA contamination. For library construction, approximately 5mg of total RNA was used to deplete ribosomal RNAs according to the Ribo-Zero rRNA Removal Kit protocol (Illumina, San Diego, United States). After removing rRNA, the remaining RNA was reverse-transcribed to generate cDNA, which was then employed to synthesize U-labeled second-stranded DNA using DNA polymerase I, RNase H, and dUTP. Thereafter, an A-base was added to the blunt ends of each strand to facilitate their ligation to the indexed adapters, which had T-base overhangs. Next, single- or dual-index adapters were ligated to the DNA molecules. After heat-labile UDG enzyme treatment of the U-labeled second-stranded DNA, the ligated products were PCR amplified under the following conditions: initial denaturation at 95°C for 3min, followed by 8cycles of denaturation at 98°C for 15s, annealing at 60°C for 15s, and extension at 72°C for 30s, with a final extension at 72°C for 5min. Next, the cDNA was gel purified and ethanol precipitated to generate a more concentrated 170–650bp (±50bp) cDNA library. Finally, paired-end sequencing on the Illumina HiSeq 4,000 platform (LC Bio, China) was performed according to the vendor’s recommended protocol.

### Bioinformatics Analysis and Prediction of Candidate sRNAs and Target Genes

After removing the adapter sequences and low-quality bases, the remaining reads were mapped to the genome (GenBank accession no. CP003499), and the reads that mapped to coding regions were filtered out. The reads that mapped to intergenic regions, regions that were complementary to known annotated genes and untranslated regions (UTRs) were searched against the Rfam database. Next, the sRNA sequences were functionally annotated, and unannotated sRNAs with a copy number of greater than 10 were selected as candidate sRNAs with the Megablast tool using the parameters of a word length of 26 and an identity percentage cutoff of 90. Target genes were predicted using RIsearch^1^ and CopraRNA.^2^ In addition, we applied a gene ontology (GO) assignment program to the predicted target genes, and functional annotation was performed using Goatools^3^ to enhance the analysis. A Kyoto Encyclopedia of Genes and Genomes pathway database (KEGG) analysis was performed to further evaluate the influence of target genes on biological/signaling pathways. Based on the differential expression of sRNAs and their target genes, we further selected those sequences that were coexpressed with a fold-change (FC) equal to or greater than |2|.

### qRT-PCR Analysis

To evaluate the expression levels of the identified sRNA genes, qRT-PCR was performed. Total RNA was isolated *via* the hot phenol method using the TRK-1002 Kit (LC Bio, China). The resulting cDNA was stored at −20°C until qRT-PCR was performed. Fourteen sRNAs were selected for verification using primers that were designed with Premier 5.0, which are shown in Table 1. qRT-PCR was carried out in 96-well plates using the ABI StepOnePlus PCR system (America). The 16S rRNA gene was used as an internal control to normalize cycle threshold (CT) values. The 2^−(ΔΔCT)^ method was used to assess the differences in the expression levels of sRNA genes. Three technical replicates were performed for each sample.

**Table 1:** The 14 selected candidate sRNAs for RT-PCR validation.

sRNA name	Start	End	Stand	Primer
sts030	354584	354318	−	5'-CAACTCATTTGAGGTTTCGCT-3'5'-AACTCTTACGCTTTAGCTGCCT-3'
sts034	925560	925631	+	5'-AAGGATTCCCAGTAGCGC-3'5'-CTTTTTTCTTTTTTAGATACACAAC-3'
sts072	2421164	2420885	−	5'-TCGCCGTTAGCTAAAGCATCTTG-3'5'-TGTGGAGGAGGTGAATCTATCCAT-3'
sts108	711756	711846	+	5'-GAGGTCATTTGCAAGATCAAG-3'5'-ATCGCTTGATCGAGATGCATGGAAA-3'
sts187	467629	467762	+	5'-TTTGAAGCGACGAAGAGCAT-3'5'-CGGAGGGAGGGAATTAACAT-3'
sts238	1141726	1141626	−	5'-TCAAATAAACAGAAGAATTTCTCGA-3'5'-CAGTGGTGAAACCTGTACTTCTAAC-3'
sts249	1548967	1548841	−	5'-TGATGGTGTAGTTGGCGTAGC-3'5'-ACAACAGTTCCTGAATCTTCAACAC-3'
sts251	1710210	1709982	−	5'-TTTCGGTGGAATGATTACTGGT-3'5'-TCCCATCTTTCAAGGAATTCTG-3'
sts347	2685474	2685367	−	5'-TCAAATAAACAGAAGAATTTCTCGA-3'5'-CAGTGGTGAAACCTGTACTTCTAAC-3'
sts392	1299103	1299271	+	5'-AGCGACCTACGAATGGGTCA-3'5'-GAACGTCCAGCAGACGACAG-3'
sts406	1830190	1830366	+	5'-GCTGGTAACACGCCTTATCAT-3'5'-CGGTGTTCGTCCTGTAGTATTG-3'
sts425	2461582	2461723	+	5'-AGTGAATGCGTATGGATGTTTG-3'5'-CCTATTCAATTTTCAGACGTTCAT-3'
sts469	1537653	1537724	+	5'-AGTCTGGATGATGGCTGATGC-3'5'-ATGCTGCAGTACTTGATGTATTGA-3'
sts472	1029846	1029748	−	5'-ATTGACGTTCAGTGGATCAA-3'5'-AGTTGAATGCTTAGTAGCTAAGC-3'

## Results

### Bioinformatic Screening Identifies sRNA Candidates in *S. thermophilus*

In the present study, 43.09 million low molecular weight RNA (<500nt) reads were obtained from all samples. Based on the read mapping and coverage statistics (Figure 1B), 68% of the reads mapped to noncoding regions, 19% of which were identified by Rfam. The remaining sequences were presumed to have mapped to candidate sRNAs. A total of 530 regulatory RNA genes were identified within the chromosomal DNA sequences of *S. thermophilus*. The accession number of sRNA was OK632712-OK633241 (see Data Sheet 1 Accession Number). RNAs in intergenic regions were annotated as sRNAs. Through small RNA sequencing, we searched for cDNA clusters that occurred specifically within intergenic regions and discovered 135 clusters that represent possible intergenic sRNA candidates, including some previously identified sRNAs, such as 6S RNA and tmRNA. RNAs that overlapped with transcripts in an antisense manner were annotated as antisense RNAs (asRNAs), which were always completely complementary to their targets. A total of 198 asRNAs were identified in the dataset. In addition to intergenic sRNAs and asRNAs, bacteria contain regulatory elements within their UTRs. These regulatory elements control transcription elongation, mRNA stability, and translation initiation in response to specific stimuli and metabolites. A total of 197 sRNAs located in untranslated mRNA regions (500nt upstream or downstream of annotated genes) were found in the chromosome of *S. thermophilus*.

### Analysis of the Expression Levels of sRNA Genes in Different Growth Phases

**Figure 2:**
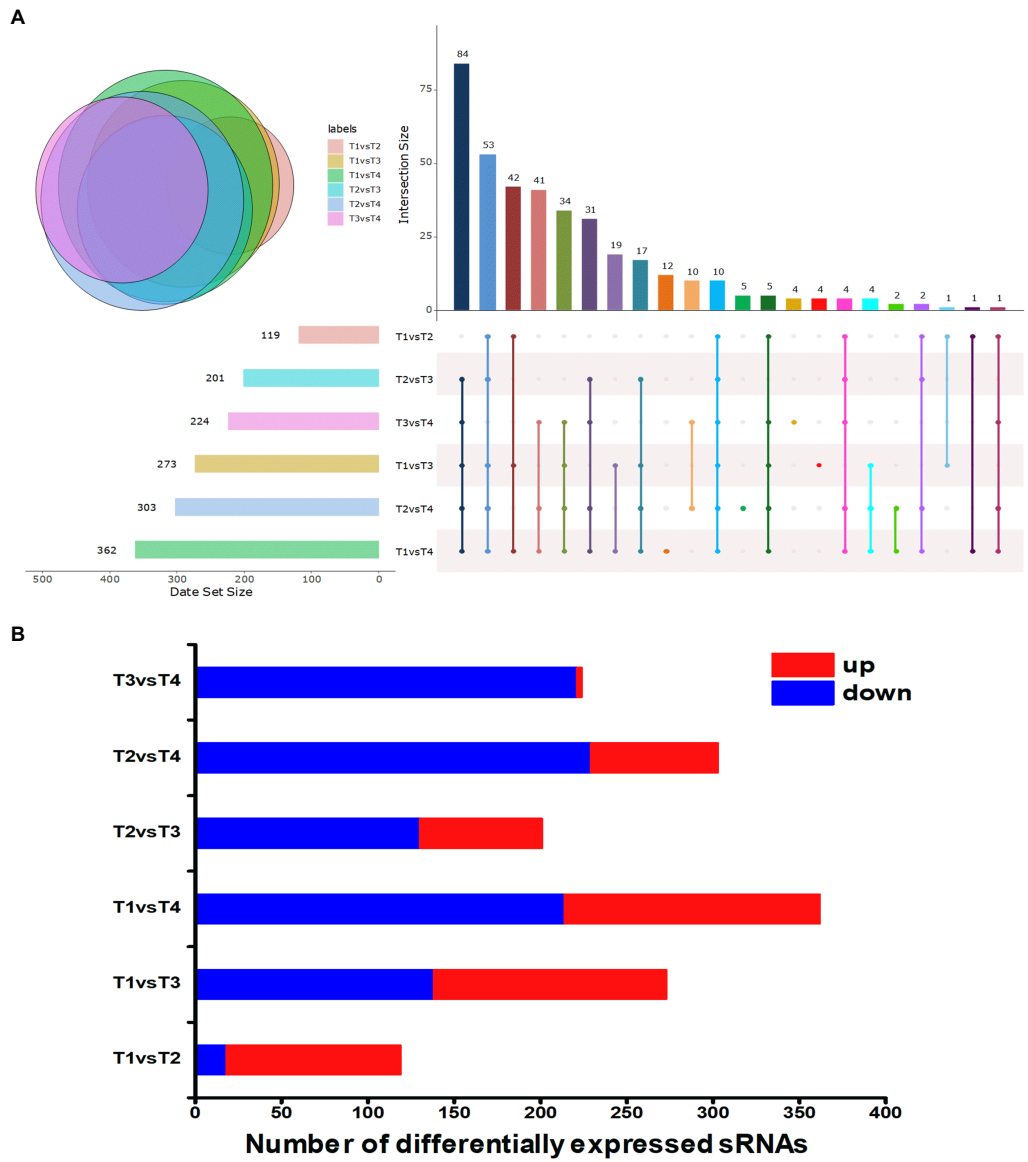
**(A)** Overlapping analysis diagram of differentially expressed sRNAs. T1, T2, T3, and T4 indicate the late-lag (early-exponential), mid-exponential, late-exponential, and stationary phases, respectively. **(B)** Statistical graph of differentially expressed sRNAs. The *x* axis represents the number of differentially expressed sRNAs. Red represents upregulated sRNA, and blue represents downregulated sRNA. T1, T2, T3 and T4 indicate the late-lag (early-exponential), mid-exponential, late-exponential, and stationary phases, respectively.

To gain insight into the expression patterns of the identified sRNA genes, those that exhibited altered expression patterns (fold change ≥|2| and *p*≤0.01) during each growth phase were visualized in a heat map (Figure 1C). The expression levels of sRNA genes are shown in Supplementary Data Table S1. The differential expression levels of sRNA in different growth phases of *S. thermophilus* were compared and analyzed (Figure 2A). Compared with the end of the lag phase and the middle of the exponential growth phase, there were large numbers of differentially expressed sRNAs at the end of the exponential growth phase and the stable phase. The overlap analysis of differentially expressed sRNAs in different periods showed that the expression levels of 10 sRNAs (sts115, sts166, sts238, sts245, sts249, sts258, sts340, sts344, sts389, and sts482) were significantly different in the six comparison groups. The number of significantly upregulated and downregulated sRNAs between the comparison groups in different growth phases is shown in Figure 2B. The results showed that compared with the end of the lag phase, most of the sRNAs were significantly upregulated in the middle of the exponential growth phase. The number of significantly upregulated and significantly downregulated sRNAs accounted for half at the end of the exponential growth phase. Approximately 60% of the sRNAs were significantly downregulated in the stationary phase.

### Functional Categorization of Candidate sRNA Target Genes

**Figure 3:**
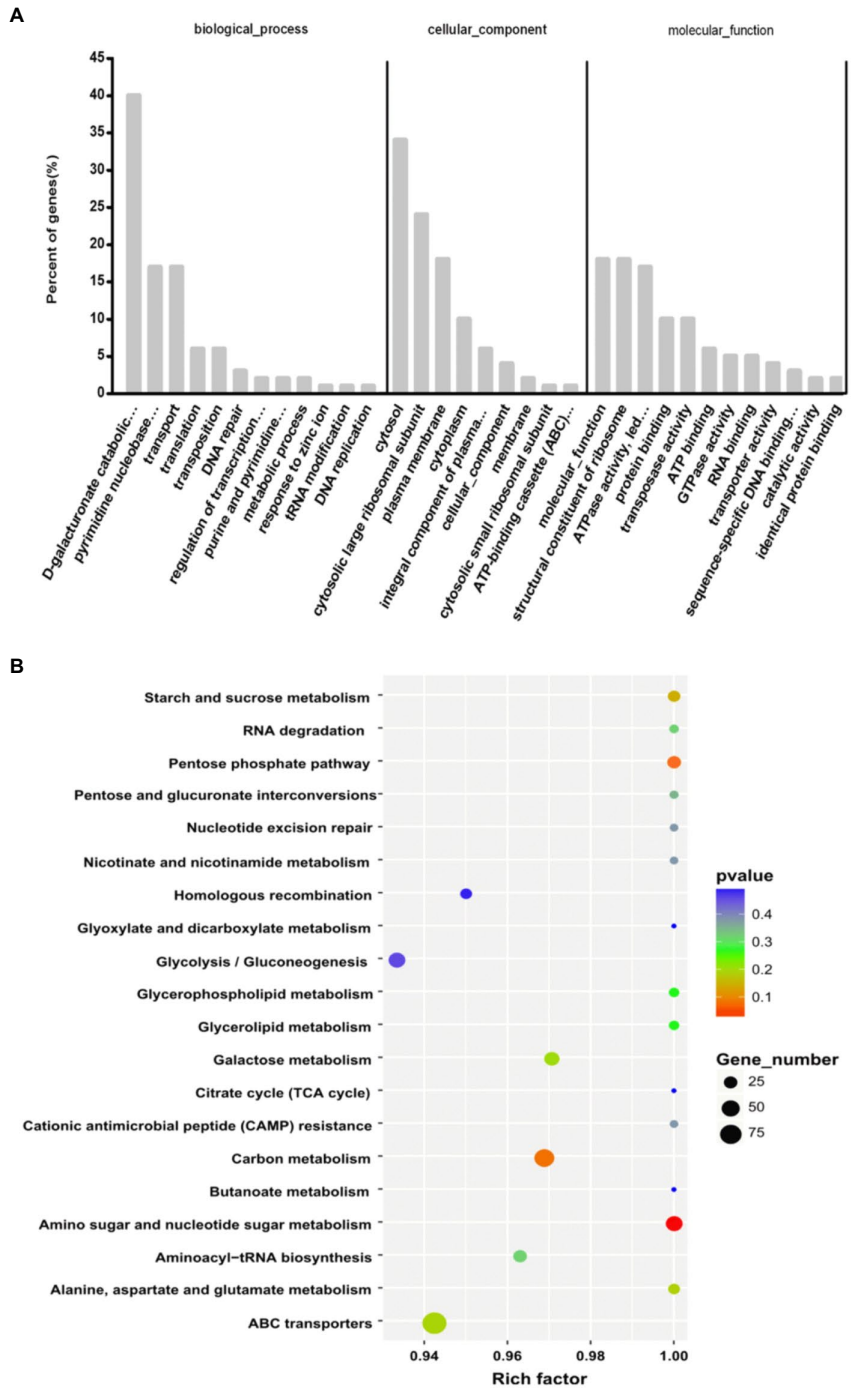
Functional analyses of predicted target sRNA genes. **(A)** GO analysis of the target genes predicted to be involved in biological processes, molecular functions and their cellular components. **(B)** KEGG analysis of target genes participating in major pathways.

To better understand the functions of sRNAs in different biological modules, target genes were predicted. A total of 390 target genes were predicted for 530 sRNAs. Enriched GO terms were searched through GO enrichment analysis and defined by the functional significance of their target genes. According to the results of a GO assignment analysis, shown in Figure 3A, the GO terms were widely distributed with their respective biological processes. D-galacturonate catabolic process and pyrimidine nucleobase metabolic process were the major sRNA targets. Other target genes were associated with growth regulatory factors, transporter activity, translation, DNA repair, regulation of transcription from the RNA polymerase II promoter and purine and pyrimidine biosynthetic processes. In the cellular components category, the majority of predicted targets were involved in the cytosol, plasma membrane and cytoplasm, indicating that sRNAs are closely related to different cellular structure systems. The molecular functions, structural constituents of the ribosome, and ATP activity of most of the enriched GO terms indicated that the crucial roles of sRNAs were closely related to the regulation of inheritance and metabolism.

The KEGG results shown in Figure 3B indicate that 55 genes were related to the metabolism of amino acids; 38 genes were related to carbon metabolism; 45 genes were related to ABC transporters; 40 genes were related to amino sugar and nucleotide sugar metabolism; 25 genes were related to aminoacyl-tRNA biosynthesis; and 39 genes were related to purine metabolism. Additionally, genes associated with fatty acid biosynthesis, the phosphotransferase system and two-component systems were indicated.

### Expression Levels of sRNAs, Target Genes, and Proteins Involved in Osmotic Stress, Acid Stress, and Starvation Stress

**Figure 4:**
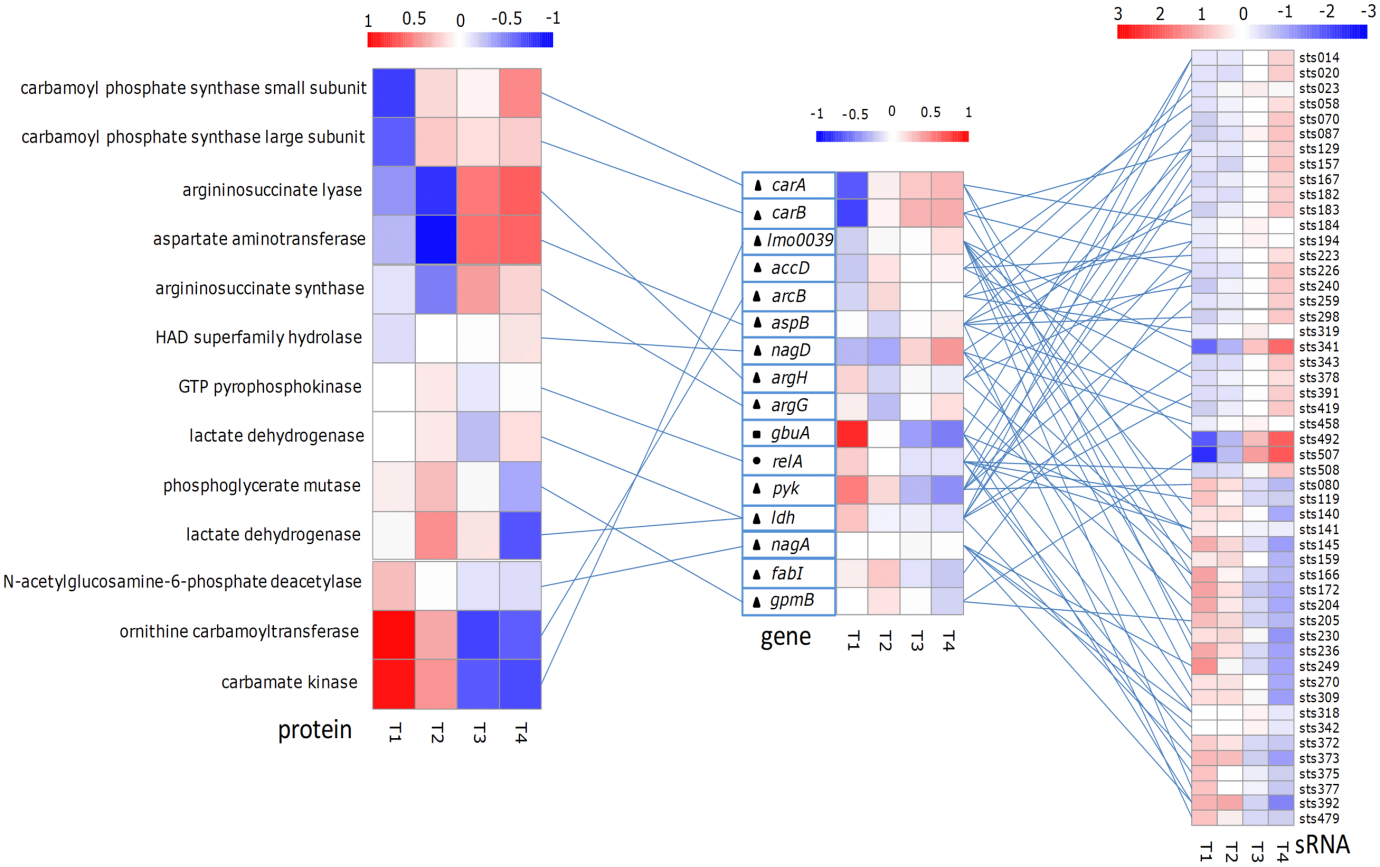
Some *Streptococcus thermophilus* proteins associated with starvation stress-, acid stress- and osmotic stress-related sRNAs, and their target differential expression heat map. The left panel shows the protein expression heat map; the middle panel shows the sRNA-targeted gene expression heat map; and the right panel shows the sRNA expression heat map. The color associated with gene and protein expression ranges from low (blue) to high (red). Heat map on the left: each column represents a library listed below, and each row represents a protein. Middle heat map: each column represents the next library, and each row represents a gene. Right side panel: each column represents the library listed below, and each row represents one sRNA. T1, T2, T3 and T4 indicate the late-lag (early-exponential), mid-exponential, late-exponential, and stationary phases, respectively. Circles, squares and triangles indicate genes involved in starvation stress, osmotic stress and acid stress, respectively. The lines between protein, gene and sRNA show the expected interactions.

In this study, we analyzed the function of the predicted target genes of putative sRNAs. According to the results, we selected some of the differentially expressed sRNAs and target genes that may be involved in osmotic stress, acid stress, and starvation stress. sRNAs, target genes, and proteins involved in osmotic stress, acid stress, and starvation stress were analyzed (Figure 4). The expression levels of these sRNAs, target genes, and proteins are shown in Supplementary Data Table S1. According to the results, the expression levels of sRNAs sts141, sts392, and sts318 involved in the regulation of osmotic stress gradually decreased at the four growth time points, whereas sRNA sts014 was gradually upregulated. The expression level of the *gbuA* gene (encoding glycine betaine/L-proline ABC transporter ATP-binding protein) coregulated by these sRNAs showed a downward trend at the four growth time points. sRNAs sts508 and sts087, which are involved in the regulation of starvation stress, were gradually upregulated at the four growth time points; however, the expression levels of sts372, sts141, sts375, and sts119 showed a downward trend. The expression levels of the target gene *relA* coregulated by these sRNAs had a trend similar to that of the *gbuA gene*. GTP pyrophosphokinase encoded by the *relA* gene had the highest expression level at the middle of the exponential growth phase and the lowest expression level at the end of the exponential growth phase. sRNAs sts129 and sts226, which are involved in arginine synthesis, were upregulated at the four growth time points. However, sRNAs sts166, sts231, sts204, sts145, and sts236 were gradually downregulated. Their target genes *carA* and *carB* were upregulated, whereas *argH* and *argG* were downregulated. The expression levels of carbamoyl phosphate synthase small subunit and carbamoyl phosphate synthase subunit encoded by *carAB* increased with increasing fermentation time; aspartate aminotransferase and argininosuccinate synthase had the lowest expression levels at the middle of the exponential growth phase. At the four growth time points, sRNAs sts033, sts341, and sts492, which are involved in the ADI pathway, were gradually upregulated; the expression levels of sRNAs sts140 and sts230 were highest at the middle of the exponential growth phase; and sRNAs sts172 and sts377 were downregulated. The target gene *lmo0039* coregulated by these sRNAs was upregulated. However, the expression level of carbamate kinase regulated by the *lmo0039* gene decreased with increasing fermentation time. Participating in acid stress regulation of sRNA can regulate the expression of multiple target genes, and these genes can also be regulated by multiple sRNAs.

### Expression Levels of sRNAs and Target Genes in Different Growth Periods

**Figure 5:**
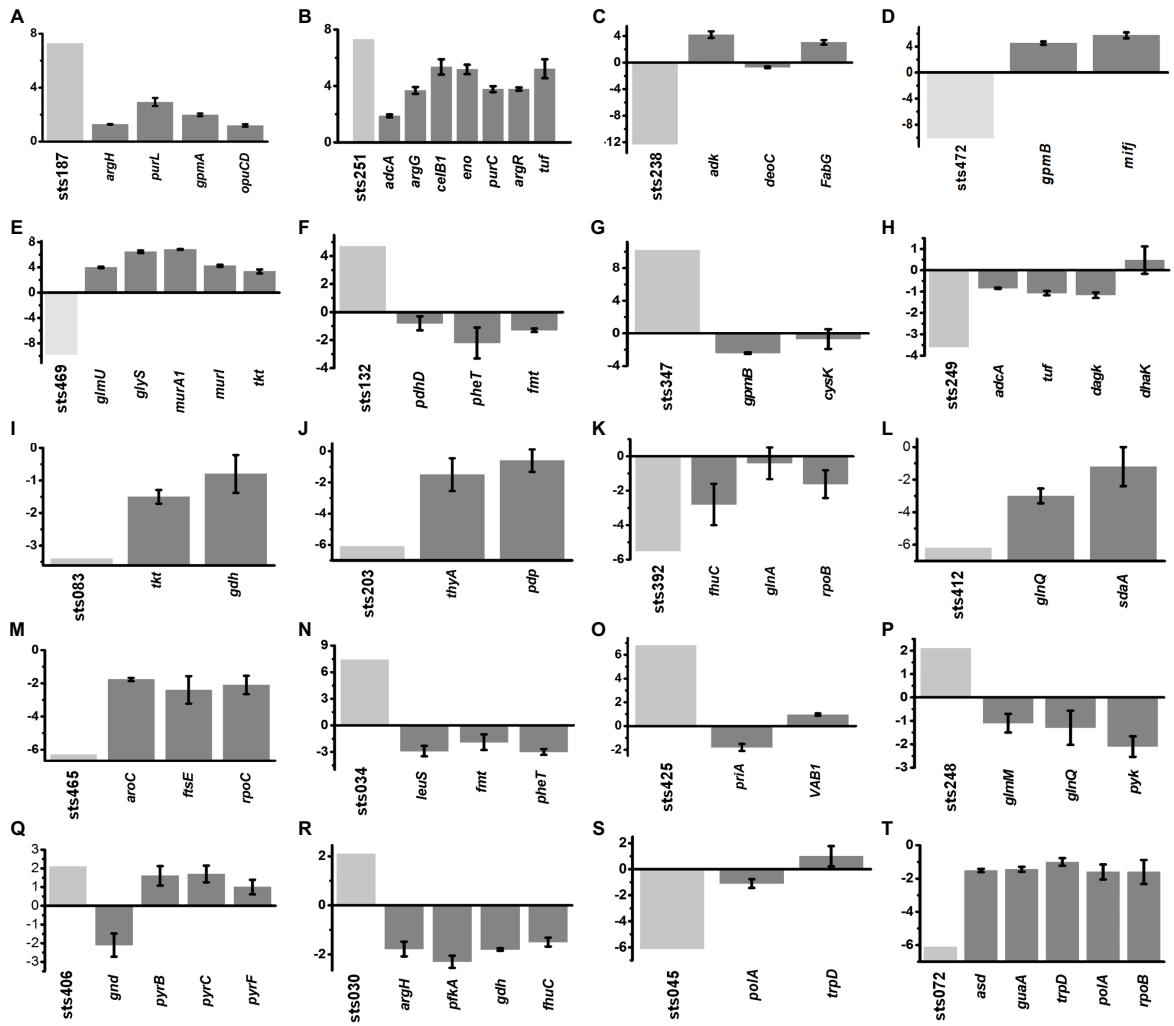
**(A-T)** Correlation between the expression of sRNAs and their target genes. The expression levels of the sRNAs (light gray) that were significantly changed in each growth phase were compared with the expression levels of the corresponding target genes (gray). Error bars indicate the mean±SD of three biological and three technical replicates.

According to the putative sRNA expression profile, we selected a total of 20 differentially expressed sRNAs from different growth stages and analyzed the expression levels of the predicted target genes of these sRNAs. The expression levels of significantly changed sRNAs and their target genes in each growth phase are shown in Figure 5. In the lag phase, 238 differentially expressed sRNA genes were observed, including 129 upregulated and 109 downregulated genes. For example, sts187 (7.3-fold) and sts251 (7.3-fold) were significantly upregulated genes whose target genes were involved in ABC transporters and the PTS system (*opuCD*, *adcA*, *celB1*, *ecfA1*), carbon metabolism (*eno*, *gmpA*), biosynthesis of arginine (*argG*, *argH*, *argR*), and purine metabolism (*purC*, *purL*), and all of their target genes were upregulated (Figure 5). From the early exponential to the mid-exponential growth phase, 84 differentially expressed sRNA genes were identified, including 64 upregulated and 18 downregulated sRNAs. Among these sRNAs, the most significantly upregulated sRNAs were sts132 (4.7-fold) and sts347 (4.1-fold). Their predicted target genes were downregulated and generally functioned in the following pathways: biosynthesis of cysteine (*cysK*), aminoacyl-tRNA biosynthesis (*fmt*, *pheT*), and glycolysis/gluconeogenesis (*gpmB*, *pdhD*). All of their target genes were downregulated (Figure 5). The most downregulated sRNAs were sts249 (−3.6-fold) and sts083 (−3.4-fold). The expression of most of the target genes of these sRNAs was also downregulated; these target genes mainly functioned as ABC transporters (*adcA*), in glycerolipid metabolism (*dagK*, *dhaK*) and in the pentose phosphate pathway (*tkt*, *gdhIV*), and as a translation elongation factor Tu (*tuf*), and all of the target genes except for *dhaK* were downregulated (Figure 5).

From the mid-exponential to the late-exponential growth phases, 193 sRNAs were differentially expressed. The expression of four sRNA genes was downregulated more than 6-fold, including sts203 (−6.1-fold), sts392 (−6.3-fold), sts412 (−6.2-fold), and sts465 (−6.3-fold). The target genes of the following functions were predicted: purine and pyrimidine metabolism (*pdp*, *thyA*, *rpoB*, *rpoC*), ABC transporters (*fhuC*, *ftsE*, *glnQ*), serine metabolism (*sdaA*), biosynthesis of tyrosine and tryptophan (*aroC*), and glutamate metabolism (*glnA*; Figure 5). All of these were downregulated. Among the upregulated sRNA genes, 26 genes showed increases of over 6.0-fold, with seven exhibiting increases of approximately 7-fold, such as sts034 (7.4-fold) and sts425 (6.8-fold). The expression of most target genes was downregulated and distributed among functions such as aminoacyl-tRNA biosynthesis (*leuS*, *fmt*, *pheT*), homologous recombination (*priA*), and oxidative phosphorylation (*VAB1*; Figure 5).

During the stationary phase, 139 sRNA genes were differentially expressed, but only three were upregulated: sts248 (2.1-fold), sts406 (2.1-fold) and sts030 (2.1-fold). The target genes of these sRNAs were commonly associated with glycolysis/gluconeogenesis (*pfkA*, *pyk*, *gnd*), ABC transporters (*fhuC*, *glnQ*), glutathione metabolism (*gdh*), biosynthesis of arginine (*argH*), amino sugar and nucleotide sugar metabolism (*glmM*), and pyrimidine and purine metabolism (*pyrB*, *pyrC*, *pyrF*), and all of the genes except *pyrB*, *pyrC*, and *pyrF* were downregulated (Figure 5). Among the downregulated sRNAs, the expression of 19 sRNAs was decreased by at least 6-fold. The target genes of sts045 (−6.1-fold), sts072 (−6.1-fold) and sts108 (−6.2-fold) were associated with the biosynthesis of amino acids (*asd*, *trpD*) and purine metabolism (*guaA*, *polA*, *rpoB*), all of which were downregulated (Figure 5).

In addition, 10 genes were selected and verified them with RNApredator. These genes are sts034, sts072, sts187, sts238, sts248, sts249, sts251, sts347, sts392, and sts425. The gene name of the predicted target gene, the pairing energy between each sRNA and its predicted target, and the gene annotation are shown in Supplementary Data Table S2.

### Identification of sRNAs and Validation by qRT-PCR

**Figure 6:**
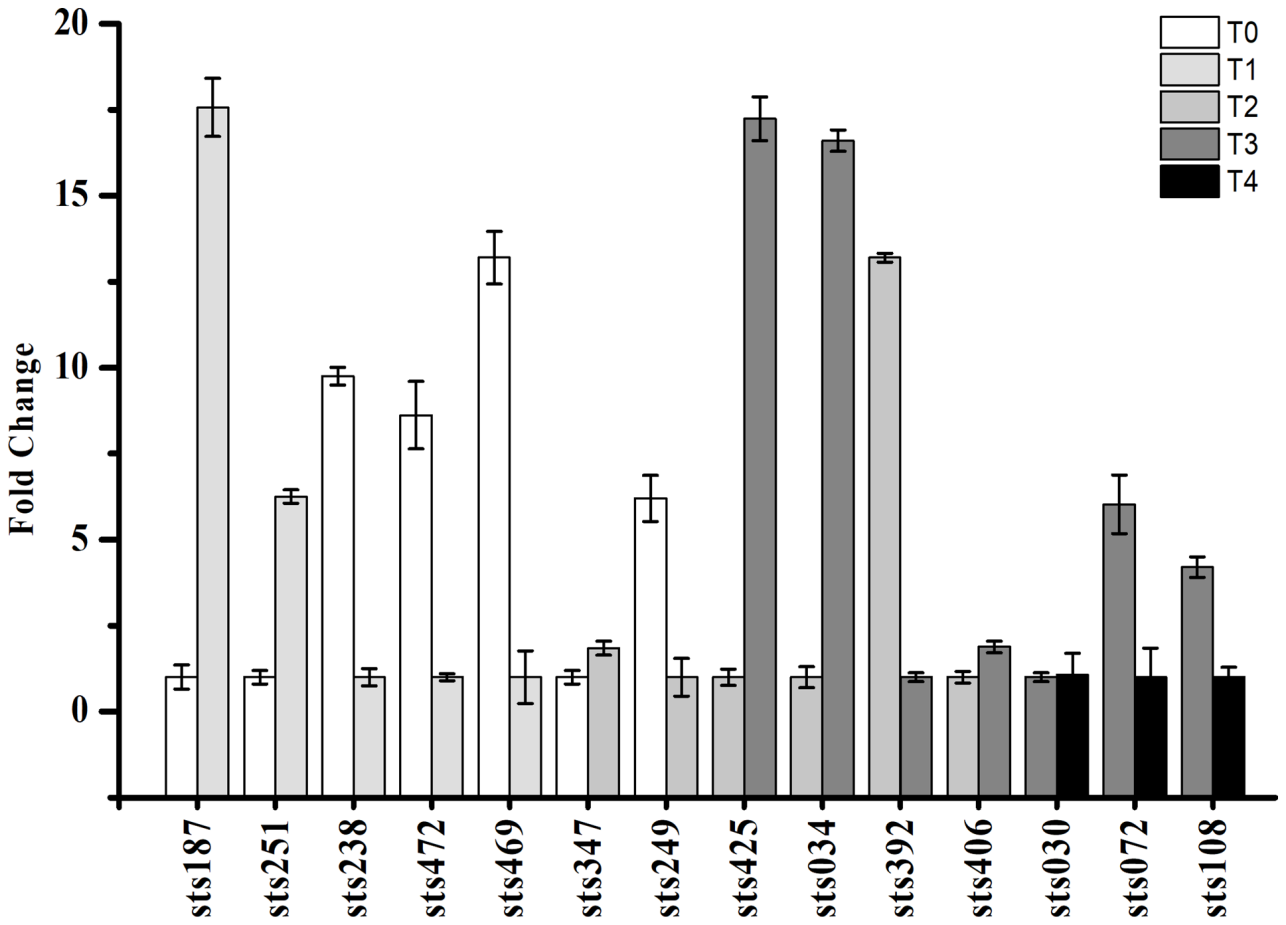
Validation of the expression of selected candidate sRNAs by qRT-PCR. The expression profiles of selected sRNA genes were assessed using the 2^−(ΔΔCT)^ method. T0, T1, T2, T3, and T4 indicate the early-lag, late-lag (early-exponential), mid-exponential, late-exponential, and stationary phases, respectively.

Small RNA-sequencing analysis revealed many novel transcribed regions, identifying 530 intergenic, antisense and UTR sRNAs. Fourteen sRNAs with significant expression levels were randomly selected for verification and identification by qRT-PCR analysis. The results are shown in Figure 6 and matched our predictions based on the sRNA-Seq results. Information on these sRNAs is shown in Table 1. According to the results, sRNA sts238, sts469, sts472, and sts238 had higher expression levels in the early lag phase. sRNA sts187 and sts251 had higher expression levels at the end of the lag phase (early exponential growth phase). sRNA sts392 had a higher expression level in the middle of the exponential growth phase. sRNA sts425, sts034, sts406, sts072, and sts108 had higher expression levels at the end of the exponential growth phase.

## Discussion

The extensive physiological loops in bacteria are regulated by a large number of sRNAs to adapt to changing conditions in the environment. Bacteria show many general and specific molecular responses to environmental changes. Sudden changes in the environment may result in changes in physical or chemical properties and may threaten the lifespan of microbial cells, especially if the stress conditions are too intense in time or intensity (van der Meulen et al., 2017). In the process of industrial fermentation, bacteria are subjected to various environmental stresses, including heat/cold shock, oxidative stress, starvation stress, osmotic stress, and low pH conditions (Romeo et al., 2007; Wang et al., 2015, 2016; Zere et al., 2015). To cope with huge environmental fluctuations, microorganisms have evolved various mechanisms to maintain cell homeostasis. sRNA has been shown to play an important role under various stress conditions in other bacteria (Gottesman et al., 2006; Romby and Charpentier, 2010; Hoe et al., 2013), and it plays a vital role in regulating growth, adaptation and survival through posttranscriptional control of gene expression in bacterial cells (Wagner and Romby, 2015; van der Meulen et al., 2017). Under stress conditions, sRNAs play a vital role in the adaptation and survival of bacteria, and a large number of sRNAs are regulated by base pairing with their target mRNA. Environmental factors such as temperature, stress, oxygen content, and nutrient deficiency can change the expression of sRNA, which in turn affects the intensity of sRNA regulation of mRNA and thus affects the growth and metabolism of bacteria at the transcription and translation levels. For example, sRNAs ArcZ, DsrA, and RprA contribute to acid tolerance in *Escherichia coli* and induce DsrA and RprA under acid stress (Bak et al., 2014).

To date, only a few sRNAs of LAB have been investigated. However, global changes at the sRNA transcription levels have not been investigated during fermentation of *S. thermophilus*. The present study analyzed the differential expression of sRNA genes during each growth phase and obtained the expression profiles. The present results lay a foundation for future studies on the physiological roles of sRNAs in *S. thermophilus*.

During the lag phase, the intracellular physiological status of cells must be adjusted to allow them to quickly adapt to the new nutritional environment and grow better during the exponential growth phase (Rault et al., 2009; Saramago et al., 2014). sRNAs were the most highly expressed and always promoted the expression of their target genes. The translation of DNA into mRNA is the first step in the synthesis of various enzymes, which promotes various metabolic activities. Genes related to nucleotide metabolism were the most highly expressed. A similar phenomenon was observed in *L. lactis* subsp. *lactis* CNRZ 157, and the expression levels of genes associated with nucleotide metabolism in the lag phase were higher than those in the exponential phase and stationary phase (Larsen et al., 2006). The intracellular pH of *S. thermophilus* must be adjusted to allow the cells to grow better during the exponential growth phase. Arginine metabolism is one of the most effective ways to regulate the intracellular acidity of LAB (Liu et al., 2018). The *argGHR* gene, encoding argininosuccinate synthase, argininosuccinate lyase and arginine regulator, is related to arginine metabolism, which can contribute to the regulation of intracellular acidic acid (Senouci-Rezkallah et al., 2011). In this study, the sRNAs sts187 and sts251, which regulate *argR*, were the most highly expressed sRNAs. The expression levels of sRNA sts204, which regulates *argG*, and sRNA sts166, which regulates *argH*, were the highest in this growth stage during the entire culture time. The argGHR gene also had higher expression levels in this growth stage. sRNA can improve the acid resistance of *S. thermophilus* by regulating the expression of *argGHR*. A similar finding was obtained in *L. lactis* F44 (Wu et al., 2018). This result indicated that sRNA could have a positive impact on energy production. sRNAs sts187 and sts251 were significantly upregulated. Their target genes (*ecfA1*, *opuCD*, *adcA*, *celB1*) related to biotin, osmoprotectants, ABC transporters and the PTS system were upregulated, indicating that sRNA can promote the absorption of nutrients by bacteria by regulating the expression of genes involved in the transport system. Our previous work produced similar findings with respect to the nutrient and energy requirements of *S. thermophilus* (Saramago et al., 2014).

From the early exponential growth phases to mid-exponential growth phases, LAB adapted to the changing environment, and the growth rate and metabolic activity gradually increased. The expression of sRNA did not change a great deal in this phase. However, the present results showed that some sRNAs inhibited the expression of target genes. The target genes of sts132 and sts347 were mainly related to aminoacyl-tRNA biosynthesis and glycolysis/gluconeogenesis. The expression levels of these target genes were downregulated. This result suggested that sRNAs could regulate translation and energy metabolism to control the rate of intracellular metabolism (Jain et al., 2012). The energy generated and the nutrients consumed are used for the growth of LAB, while at the same time, a certain amount of metabolic waste is generated and accumulates (Dutta and Srivastava, 2018). Thus, these sRNAs might be associated with the regulation of LAB metabolism to prevent the needless generation of ATP or nutrient uptake. The *cysK* gene, a target gene of sRNA sts347, whose product catalyzes the conversion between serine and cysteine, was downregulated in the early exponential growth phase and was significantly upregulated in the lag phase. This could result from intracellular concentrations of serine and cysteine meeting the growth requirements.

From the mid-exponential growth phase to the late-exponential growth phase, the cells encountered additional stress in the form of intracellular acidity and extracellular sodium lactate, which can significantly affect the growth of cells (Lengeler, 1993). sRNA can be induced by environmental changes (Siqueira et al., 2016) and serves as a key regulator of the stress response and virulence (Bardill et al., 2011; Wang et al., 2015, 2016; Zere et al., 2015). sRNA GadY can improve the growth of *E. coli* at pH 6.0 by inactivating the expression of the glutamate decarboxylase system in the late exponential growth phase. The expression of GadY will also reduce the production of acetate, thereby reducing the inhibitory effect of this acid on bacterial growth (Negrete and Shiloach, 2015). The simultaneous overexpression of sRNA DsrA and sRNA chaperone Hfq activates RpoS and significantly increases cell acid tolerance and cell survival upon extreme acid shock (Lin et al., 2021). According to the results, numerous sRNA genes were expressed in this growth phase. LAB could improve acid tolerance *via* an electrogenic Na^+^/H^+^ antiport system, and H^+^/Na^+^ symporters could promote the absorption of amino acids (Efiok and Webster, 1990). In our previous work, intracellular H^+^-ATPase activity gradually increased from the mid-exponential growth phase to the stationary phase (Liu et al., 2018). In this study, the expression of sts425 was upregulated (6.8-fold) and promoted the upregulation of the *VAB1* gene, which encodes the H^+^/Na^+^-transporting ATPase subunit. This result indicated that sRNA could have a positive effect on the H^+^/Na^+^ transport systems. Preliminary work showed that arginine, a functional nutrient component, enhanced the acid tolerance of *S. thermophilus* during the pH-controlling fermentation process and increased the biomass of *S. thermophilus* (Liu et al., 2018). The genes that regulate arginine to improve the acid tolerance of bacterial cells are the same as some of the target genes that are predicted by sRNAs regulated by bioinformatics in this study. The expression of sRNAs sts129, sts226, sts087 and sts289 increased with increasing culture time. Moreover, their target genes had higher expression levels at the end of the exponential growth period than at the middle of the exponential growth period. The expression of proteins involved in these genes, such as aspartate aminotransferase, argininosuccinate lyase, and argininosuccinate synthase, was upregulated. The results indicate that functional nutrient components may regulate the expression of target genes through sRNAs, thereby regulating protein expression to improve the acid tolerance of bacterial cells (Shan et al., 2016). In addition, the study found that while sts129 positively regulates the *carB* gene, it also has a negative regulatory effect on the *pyk* gene. sRNA can simultaneously positively and negatively regulate different genes. The expression levels of sts034 and sts425 exhibited the greatest change. The target genes of these two sRNAs were associated with aminoacyl-tRNA biosynthesis, alanine, aspartate, glutamate, cysteine, and glycine metabolism, ABC transporters and carbon metabolism and were downregulated, which could be due to a lack of sufficient nutrients in the medium to maintain growth. These results indicated that metabolic activity was decreased in this phase.

During the stationary phase, intracellular and extracellular stresses as well as a lack of sufficient nutrients inhibit the growth of LAB, such that the growth of cells nearly stops, and their intracellular metabolism decreases. The various genes of *S. thermophilus* were affected under these stress conditions. When nutrients are insufficient in the growth environment, bacterial sRNA can also play an important regulatory role in resisting nutritional stress (carbon source and nitrogen source). In *E. coli*, sRNA CsrB and CsrC can simultaneously regulate carbon metabolism and amino acid metabolism. The expression levels of sRNA CsrB and CsrC are significantly upregulated in a low-nutrient environment. When amino acids are added to a low-nutrient environment, the expression level of CsrB is significantly downregulated (Pourciau et al., 2020). sRNA can also regulate the use of some trace elements by bacteria by regulating the expression of target genes, such as those involved in iron homeostasis (Oglesby et al., 2010; Holmqvist and Wagner, 2017). In this study, sRNAs sts030, sts248, and 6S RNA (sts406) were upregulated. In *L. lactis*, the expression of 6S RNA was carbon source-dependent, which had a positive effect on balancing nutritional demands during the stationary phase (Storz et al., 2005; Wassarman, 2007; van der Meulen et al., 2017). Three upregulated target genes of sts406, encoding pyrimidine biosynthetic proteins (*PyrBCF*), indicated that the requirements of pyrimidine nucleotides increased in this phase. sRNA sts406 could regulate pyrimidine and purine metabolism to maintain the growth of LAB. An analysis of other upregulated sRNA target genes showed that they had a negative influence on the biosynthesis of amino acids, ABC transporters and purine metabolism. In the absence of nutrients, bacteria maintain their own activity by regulating gene expression to reduce nutrient waste. During starvation, the signal molecules ppGpp and pppGpp [together called (p)ppGpp] accumulate in the bacteria, regulating metabolism, virulence and the growth rate, switching the metabolic balance from growth and cell division to survival and stress response, and playing an important role in resistance to adversity (Sanyal and Harinarayanan, 2020; Kurata et al., 2021; May et al., 2021). The *relA* gene synthesizes (p)ppGpp in response to amino acid restriction (Sinha and Winther, 2021). In our previous studies, most of the amino acids in CDM were consumed in large amounts during the lag phase, especially arginine, whose consumption at this stage accounted for approximately 99.75% of the total arginine consumption in the medium. In this growth phase, the expression level of *relA* was the highest, which may be related to the high consumption of amino acids. Except for sts502, the expression levels of other sRNAs that regulate the *relA* gene were downregulated. The expression of the target gene *relA* also gradually decreased with culture time. This may be because the bacteria accumulated enough (p)ppGpp and gradually adapted to amino acid starvation. Furthermore, the expression of sRNAs involved in osmotic stress regulation and the target genes of these sRNAs decreased with increasing incubation time.

## Conclusion

The expression profiles of sRNAs in *S. thermophilus* were analyzed, and 530 candidate sRNAs were identified. The results suggest that sRNAs are involved in several metabolic pathways, particularly in amino acid metabolism and carbon metabolism. Furthermore, sRNAs play an important role in the regulation of starvation stress, acid stress, and osmotic stress by regulating target gene expression during the culture of *S. thermophilus*. These results indicate a future research direction for sRNA in *S. thermophilus*. The present study also lays a foundation for future studies on the functions of sRNAs. Overall, our results provide new insights for understanding the complex regulatory network of sRNAs in *S. thermophilus*.

## Data Availability Statement

The datasets presented in this study can be found in online repositories. The names of the repository/repositories and accession number(s) can be found in the article/Supplementary Material.

## Author Contributions

GL, YZ, and ZF designed the experiments. GL, HC, and YQ carried out the experimental work. HC, YZ, and AZ participated in the sample collection. GL, YZ, and HC analyzed and interpreted the data. GL wrote the manuscript. GL, YZ and ZF advised on the manuscript content. All authors contributed to the article and approved the submitted version.

## Funding

This work was supported by grants from the National Natural Science Foundation of China (31771989) and the Open Research Fund for Key Laboratory of Dairy Science (Northeast Agricultural University), Ministry of Education, Harbin China (2020-KLDS-OF-004).

## Conflict of Interest

The authors declare that the research was conducted in the absence of any commercial or financial relationships that could be construed as a potential conflict of interest.

## Publisher’s Note

All claims expressed in this article are solely those of the authors and do not necessarily represent those of their affiliated organizations, or those of the publisher, the editors and the reviewers. Any product that may be evaluated in this article, or claim that may be made by its manufacturer, is not guaranteed or endorsed by the publisher.

## Footnotes

^1^ http://rth.dk/resources/risearch

^2^ http://rna.informatik.uni-freiburg.de/CopraRNA/Input.jsp

^3^ https://github.com/tanghaibao/GOatools

## References

[ref1] Andersen J. M. Pedersen C. M. Bang-Berthelsen C. H. (2019). Omics-based comparative analysis of putative mobile genetic elements in *Lactococcus lactis*. FEMS Microbiol. Lett. 366(Suppl. 1), i105–i113. doi: 10.1093/femsle/fnz102s 31074793

[ref2] Bak G. Han K. Kim D. Lee Y. (2014). Roles of rpoS-activating small RNAs in pathways leading to acid resistance of *Escherichia coli*. Microbiology 3, 15–28. doi: 10.1002/mbo3.143, PMID: 24319011 PMC3937726

[ref3] Bardill J. P. Zhao X. Hammer B. K. (2011). The vibrio cholerae quorum sensing response is mediated by Hfq-dependent sRNA/mRNA base pairing interactions. Mol. Microbiol. 80, 1381–1394. doi: 10.1111/j.1365-2958.2011.07655.x, PMID: 21453446

[ref4] Dequan Z. Fei L. Limei S. Yang X. Xiangchen M. (2015). Genome-wide identification of small RNAs in *Bifidobacterium animalis* subsp. *lactis* KLDS 2.0603 and their regulation role in the adaption to gastrointestinal environment. PLoS One 10:e0117373. doi: 10.1371/journal.pone.0117373, PMID: 25706951 PMC4338058

[ref5] Dutta T. Srivastava S. (2018). Small RNA-mediated regulation in bacteria: a growing palette of diverse mechanisms. Gene 656:60. doi: 10.1016/j.gene.2018.02.068, PMID: 29501814

[ref6] Efiok B. J. S. Webster D. A. (1990). Respiratory-driven sodium electrical potential in the bacterium, vitreoscilla. Biochemistry 29, 4734–4739. doi: 10.1021/bi00471a030, PMID: 2372555

[ref7] Gao Y. Liu Y. Sun M. Zhang H. Tuo Y. (2020). Physiological function analysis of *lactobacillus plantarum* Y44 based on genotypic and phenotypic characteristics. J. Dairy Sci. 103, 5916–5930. doi: 10.3168/jds.2019-18047, PMID: 32418691

[ref8] Gottesman S. Mccullen C. A. Guillier M. Vanderpool C. K. Majdalani N. Benhammou J.. (2006). Small RNA regulators and the bacterial response to stress. Cold Spring Harb. Symp. Quant. Biol. 71, 1–11. doi: 10.1101/sqb.2006.71.016, PMID: 17381274 PMC3592358

[ref9] Hoe C. H. Raabe C. A. Rozhdestvensky T. S. Tang T. H. (2013). Bacterial sRNAs: regulation in stress. Int. J. Med. Microbiol. 303, 217–229. doi: 10.1016/j.ijmm.2013.04.002 23660175

[ref10] Holmqvist E. Wagner E. G. H. (2017). Impact of bacterial sRNAs in stress responses. Biochem. Soc. Trans. 45, 1203–1212. doi: 10.1042/BST20160363 29101308 PMC5730939

[ref11] Jacques J. F. Jang S. Prévost K. Desnoyers G. Desmarais M. Imlay J.. (2006). RyhB small RNA modulates the free intracellular iron pool and is essential for normal growth during iron limitation in *Escherichia coli*. Mol. Microbiol. 62, 1181–1190. doi: 10.1111/j.1365-2958.2006.05439.x, PMID: 17078818

[ref12] Jain M. Nilsson R. Sharma S. Madhusudhan N. Kitami T. Souza A. L.. (2012). Metabolite profiling identifies a key role for glycine in rapid cancer cell proliferation. Science 336, 1040–1044. doi: 10.1126/science.1218595 22628656 PMC3526189

[ref13] Kang X. Ling N. Sun G. Zhou Q. Zhang L. Sheng Q. (2012). Complete genome sequence of *Streptococcus thermophilus* strain MN-ZLW-002. J. Bacteriol. 194, 4428–4429. doi: 10.1128/JB.00740-12, PMID: 22843572 PMC3416250

[ref14] Karvelis T. Gasiunas G. Miksys A. Barrangou R. Horvath P. Siksnys V. (2013). crRNA and tracrRNA guide Cas9-mediated DNA interference in *Streptococcus thermophilus*. RNA Biol. 10, 841–851. doi: 10.4161/rna.24203, PMID: 23535272 PMC3737341

[ref15] Klein G. Raina S. (2017). Small regulatory bacterial RNAs regulating the envelope stress response. Biochem. Soc. Trans. 45, 417–425. doi: 10.1042/BST20160367 28408482 PMC5736990

[ref16] Kurata T. Brodiazhenko T. Oliveira S. Roghanian M. Hauryliuk V. (2021). RelA-SpoT homolog toxins pyrophosphorylate the CCA end of tRNA to inhibit protein synthesis. Mol. Cell 81, 3160–3170. doi: 10.1016/j.molcel.2021.06.005 34174184

[ref17] Lahtvee P. J. Adamberg K. Arike L. Nahku R. Aller K. Vilu R. (2011). Multi-omics approach to study the growth efficiency and amino acid metabolism in *Lactococcus lactis* at various specific growth rates. Microb. Cell Factories 10, 1–12. doi: 10.1186/1475-2859-10-12 PMC3049130 21349178

[ref18] Larsen N. Boye M. Siegumfeldt H. Jakobsen M. (2006). Differential expression of proteins and genes in the lag phase of *Lactococcus lactis* subsp. *lactis* grown in synthetic medium and reconstituted skim milk. Appl. Environ. Microbiol. 72, 1173–1179. doi: 10.1128/AEM.72.2.1173-1179.2006, PMID: 16461664 PMC1392913

[ref19] Lengeler J. W. (1993). Carbohydrate transport in bacteria under environmental conditions, a black box. Antonie Van Leeuwenhoek 63, 275–288. doi: 10.1007/BF00871223 8279824

[ref20] Lin Z. Li J. Yan X. Yang J. Yang X. (2021). Engineering of the sRNA DsrA together with the sRNA chaperone Hfq enhances the acid tolerance of *E. coli*. Appl. Environ. Microbiol. 87, e02923–e03020. doi: 10.1128/AEM.02923-20, PMID: 33674434 PMC8117753

[ref21] Liu G. Qiao Y. Zhang Y. Leng C. Sun J. Chen H.. (2018). Profiles of *Streptococcus thermophilus* MN-ZLW-002 nutrient requirements in controlled pH batch fermentations. Microbiology 8:e00633. doi: 10.1002/mbo3.633, PMID: 29682906 PMC6391275

[ref22] Marx P. Nuhn M. Kovács M. Hakenbeck R. Brückner R. (2010). Identification of genes for small non-coding RNAs that belong to the regulon of the two-component regulatory system CiaRH in streptococcus. BMC Genom. 11:661. doi: 10.1186/1471-2164-11-661, PMID: 21106082 PMC3091779

[ref23] Massé E. Gottesman S. (2002). A small RNA regulates the expression of genes involved in iron metabolism in *Escherichia coli*. Proc. Natl. Acad. Sci. U. S. A. 99, 4620–4625. doi: 10.1073/pnas.032066599 11917098 PMC123697

[ref24] May A. E. Jihen Z. Snoussi S. Mouhoub R. B. Landoulsi A. (2021). *RelA* and *spoT* gene expression is modulated in salmonella grown under static magnetic field. Curr. Microbiol. 3, 887–893. doi: 10.1007/s00284-021-02346-7 33515321

[ref25] Miao S. Wu H. Zhao Y. Caiyin Q. Li Y. Qiao J. (2018). Enhancing nisin yield by engineering a small noncodding RNA anti41 and inhibiting the expression of *glnR* in *Lactococcus lactis* F44. Biotechnol. Lett. 40, 941–948. doi: 10.1007/s10529-018-2550-3 29619745

[ref26] Negrete A. Shiloach J. (2015). Constitutive expression of the sRNA GadY decreases acetate production and improves *E. coli* growth. Microb. Cell Factories 14, 1–10. doi: 10.1186/s12934-015-0334-1 PMC4574537 26383169

[ref27] Oglesby A. G. Murphy E. R. Iyer V. R. Payne S. M. (2010). Fur regulates acid resistance in *Shigella flexneri* via RyhB and *ydeP*. Mol. Microbiol. 58, 1354–1367. doi: 10.1111/j.1365-2958.2005.04920.x 16313621

[ref28] Pourciau C. Lai Y. J. Gorelik M. Babitzke P. Romeo T. (2020). Diverse mechanisms and circuitry for global regulation by the RNA-binding protein CsrA. Front. Microbiol. 11:601352. doi: 10.3389/fmicb.2020.601352 33193284 PMC7652899

[ref29] Prajapati J. B. Nathani N. M. Patel A. K. Senan S. Joshi C. G. (2013). Genomic analysis of dairy starter culture *Streptococcus thermophilus* MTCC 5461. Microb. Biotechnol. 23, 459–466. doi: 10.4014/jmb.1210.10030, PMID: 23568199

[ref30] Qi J. Caiyin Q. Wu H. Tian K. Wang B. Li Y.. (2017). The novel sRNA s015 improves nisin yield by increasing acid tolerance of *Lactococcus lactis* F44. Appl. Microbiol. Biotechnol. 101, 6483–6493. doi: 10.1007/s00253-017-8399-x, PMID: 28689267

[ref31] Rault A. Bouix M. Béal C. (2009). Fermentation pH influences the physiological-state dynamics of *lactobacillus bulgaricus* CFL1 during pH-controlled culture. Appl. Environ. Microbiol. 75, 4374–4381. doi: 10.1128/AEM.02725-08, PMID: 19429565 PMC2704822

[ref32] Romby P. Charpentier E. (2010). An overview of RNAs with regulatory functions in gram-positive bacteria. Cell. Mol. Life Sci. 67, 217–237. doi: 10.1007/s00018-009-0162-8, PMID: 19859665 PMC11115938

[ref33] Romeo Y. Bouvier J. Gutierrez C. (2007). Osmotic regulation of transcription in *Lactococcus lactis*: ionic strength-dependent binding of the BusR repressor to the *busA* promoter. FEBS Lett. 581, 3387–3390. doi: 10.1016/j.febslet.2007.06.037, PMID: 17603047

[ref34] Sanyal R. Harinarayanan R. (2020). Activation of RelA by pppGpp as the basis for its differential toxicity over ppGpp in *Escherichia coli*. J. Biosci. 45, 1–13. doi: 10.1007/s12038-020-9991-2 32020910

[ref35] Saramago M. Bárria C. Dos Santos R. F. Silva I. J. Pobre V. Domingues S.. (2014). The role of RNases in the regulation of small RNAs. Curr. Opin. Microbiol. 18, 105–115. doi: 10.1016/j.mib.2014.02.009, PMID: 24704578

[ref36] Senouci-Rezkallah K. Schmitt P. Jobin M. P. (2011). Amino acids improve acid tolerance and internal pH maintenance in *Bacillus cereus* ATCC14579 strain. Food Microbiol. 28, 364–372. doi: 10.1016/j.fm.2010.09.003, PMID: 21356439

[ref37] Shan H. Zhengwen A. Xiaomei S. Gefei L. Shuang Z. Min Z.. (2016). Influence of arginine on the growth, arginine metabolism and amino acid consumption profiles of *Streptococcus thermophilus* T1C2 in controlled pH batch fermentations. J. Appl. Microbiol. 121, 746–756. doi: 10.1111/jam.13221, PMID: 27377190

[ref38] Sinha A. K. Winther K. S. (2021). The RelA hydrolase domain acts as a molecular switch for (p) ppGpp synthesis. Commun. Biol. 4, 1–10. doi: 10.1038/s42003-021-01963-z 33790389 PMC8012599

[ref39] Siqueira F. M. de Morais G. L. Higashi S. Beier L. S. Breyer G. M. de Sá Godinho C. P.. (2016). Mycoplasma non-coding RNA: identification of small RNAs and targets. BMC Genom. 17, 327–335. doi: 10.1186/s12864-016-3061-z PMC5088518 27801290

[ref40] Storz G. Altuvia S. Wassarman K. M. (2005). An abundance of RNA regulators. Annu. Rev. Biochem. 74, 199–217. doi: 10.1146/annurev.biochem.74.082803.133136, PMID: 15952886

[ref41] Tian K. Li Y. Wang B. Wu H. Caiyin Q. Zhang Z.. (2019). The genome and transcriptome of *Lactococcus lactis* ssp. *lactis* F44 and G423: insights into adaptation to the acidic environment. J. Dairy Sci. 102, 1044–1058. doi: 10.3168/jds.2018-14882 30594364

[ref42] van der Meulen S. B. de Jong A. Kok J. (2016). Transcriptome landscape of *Lactococcus lactis* reveals many novel RNAs including a small regulatory RNA involved in carbon uptake and metabolism. RNA Biol. 13, 353–366. doi: 10.1080/15476286.2016.1146855, PMID: 26950529 PMC4829306

[ref43] van der Meulen S. B. de Jong A. Kok J. (2017). Early transcriptome response of *Lactococcus lactis* to environmental stresses reveals differentially expressed small regulatory RNAs and tRNAs. Front. Microbiol. 8:1704. doi: 10.3389/fmicb.2017.01704, PMID: 28959239 PMC5603721

[ref44] Wagner E. G. H. Romby P. (2015). Small RNAs in bacteria and archaea: who they are, what they do, and how they do it. Adv. Genet. 90, 133–208. doi: 10.1016/bs.adgen.2015.05.001 26296935

[ref45] Wang J. Sun Z. Qiao J. Chen D. Cheng C. Luo X.. (2018). Metatranscriptome profiling of the dynamic transcription of mRNA and sRNA of a probiotic lactobacillus strain in human gut. bioRxiv. doi: 10.1101/442673

[ref46] Wang L. Wang W. Li F. Zhang J. Wu J. Gong Q.. (2015). Structural insights into the recognition of the internal A-rich linker from OxyS sRNA by *Escherichia coli* Hfq. Nucleic Acids Res. 43, 2400–2411. doi: 10.1093/nar/gkv072, PMID: 25670676 PMC4344510

[ref47] Wang L. Yang G. Qi L. Li X. Jia L. Xie J.. (2016). A novel small RNA regulates tolerance and virulence in *Shigella flexneri* by responding to acidic environmental changes. Front. Cell. Infect. Microbiol. 6:24. doi: 10.3389/fcimb.2016.00024, PMID: 27014636 PMC4782007

[ref48] Wassarman K. M. (2007). 6S RNA: a small RNA regulator of transcription. Curr. Opin. Microbiol. 10, 164–168. doi: 10.1016/j.mib.2007.03.008, PMID: 17383220

[ref49] Wu H. Song S. Tian K. Zhou D. Wang B. Liu J.. (2018). A novel small RNA S042 increases acid tolerance in *Lactococcus lactis* F44. Biochem. Biophys. Res. Commun. 500, 544–549. doi: 10.1016/j.bbrc.2018.04.069, PMID: 29654767

[ref50] Yuki N. Narumi S. Taihei U. Yamashita H. Yasuda S. Igoshi K.. (2020). Functions of small RNAs in *lactobacillus casei-Pediococcus* group of lactic acid bacteria using fragment analysis. FEMS Microbiol. Lett. 367:fnaa154. doi: 10.1093/femsle/fnaa154, PMID: 33068404

[ref51] Zere T. R. Vakulskas C. A. Leng Y. Pannuri A. Potts A. H. Dias R.. (2015). Genomic targets and features of BarA-UvrY (-SirA) signal transduction systems. PLoS One 10:e0145035. doi: 10.1371/journal.pone.0145035 26673755 PMC4682653

[ref52] Zorgani M. A. Quentin R. Lartigue M. F. (2016). Regulatory RNAs in the less studied *streptococcal* species: from nomenclature to identification. Front. Microbiol. 7:1161. doi: 10.3389/fmicb.2016.01161, PMID: 27507970 PMC4960207

