# Supplementary material for: Profiles of Small Regulatory RNAs at Different Growth Phases of Streptococcus thermophilus During pH-Controlled Batch Fermentation
Source: Front Microbiol. 2021 Nov 30;12:765144. doi: 10.3389/fmicb.2021.765144 (PMC8753986; doi:10.3389/fmicb.2021.765144)
Supplement: Supplementary file 3 [file Data_Sheet_1.DOCX]

BankIt2503238 sts001 OK632712

BankIt2503238 sts002 OK632713

BankIt2503238 sts003 OK632714

BankIt2503238 sts004 OK632715

BankIt2503238 sts005 OK632716

BankIt2503238 sts006 OK632717

BankIt2503238 sts007 OK632718

BankIt2503238 sts008 OK632719

BankIt2503238 sts009 OK632720

BankIt2503238 sts010 OK632721

BankIt2503238 sts011 OK632722

BankIt2503238 sts012 OK632723

BankIt2503238 sts013 OK632724

BankIt2503238 sts014 OK632725

BankIt2503238 sts015 OK632726

BankIt2503238 sts016 OK632727

BankIt2503238 sts017 OK632728

BankIt2503238 sts018 OK632729

BankIt2503238 sts019 OK632730

BankIt2503238 sts020 OK632731

BankIt2503238 sts021 OK632732

BankIt2503238 sts022 OK632733

BankIt2503238 sts023 OK632734

BankIt2503238 sts024 OK632735

BankIt2503238 sts025 OK632736

BankIt2503238 sts026 OK632737

BankIt2503238 sts027 OK632738

BankIt2503238 sts028 OK632739

BankIt2503238 sts029 OK632740

BankIt2503238 sts030 OK632741

BankIt2503238 sts031 OK632742

BankIt2503238 sts032 OK632743

BankIt2503238 sts033 OK632744

BankIt2503238 sts034 OK632745

BankIt2503238 sts035 OK632746

BankIt2503238 sts036 OK632747

BankIt2503238 sts037 OK632748

BankIt2503238 sts038 OK632749

BankIt2503238 sts039 OK632750

BankIt2503238 sts040 OK632751

BankIt2503238 sts041 OK632752

BankIt2503238 sts042 OK632753

BankIt2503238 sts043 OK632754

BankIt2503238 sts044 OK632755

BankIt2503238 sts045 OK632756

BankIt2503238 sts046 OK632757

BankIt2503238 sts047 OK632758

BankIt2503238 sts048 OK632759

BankIt2503238 sts049 OK632760

BankIt2503238 sts050 OK632761

BankIt2503238 sts051 OK632762

BankIt2503238 sts052 OK632763

BankIt2503238 sts053 OK632764

BankIt2503238 sts054 OK632765

BankIt2503238 sts055 OK632766

BankIt2503238 sts056 OK632767

BankIt2503238 sts057 OK632768

BankIt2503238 sts058 OK632769

BankIt2503238 sts059 OK632770

BankIt2503238 sts060 OK632771

BankIt2503238 sts061 OK632772

BankIt2503238 sts062 OK632773

BankIt2503238 sts063 OK632774

BankIt2503238 sts064 OK632775

BankIt2503238 sts065 OK632776

BankIt2503238 sts066 OK632777

BankIt2503238 sts067 OK632778

BankIt2503238 sts068 OK632779

BankIt2503238 sts069 OK632780

BankIt2503238 sts070 OK632781

BankIt2503238 sts071 OK632782

BankIt2503238 sts072 OK632783

BankIt2503238 sts073 OK632784

BankIt2503238 sts074 OK632785

BankIt2503238 sts075 OK632786

BankIt2503238 sts076 OK632787

BankIt2503238 sts077 OK632788

BankIt2503238 sts078 OK632789

BankIt2503238 sts079 OK632790

BankIt2503238 sts080 OK632791

BankIt2503238 sts081 OK632792

BankIt2503238 sts082 OK632793

BankIt2503238 sts083 OK632794

BankIt2503238 sts084 OK632795

BankIt2503238 sts085 OK632796

BankIt2503238 sts086 OK632797

BankIt2503238 sts087 OK632798

BankIt2503238 sts088 OK632799

BankIt2503238 sts089 OK632800

BankIt2503238 sts090 OK632801

BankIt2503238 sts091 OK632802

BankIt2503238 sts092 OK632803

BankIt2503238 sts093 OK632804

BankIt2503238 sts094 OK632805

BankIt2503238 sts095 OK632806

BankIt2503238 sts096 OK632807

BankIt2503238 sts097 OK632808

BankIt2503238 sts098 OK632809

BankIt2503238 sts099 OK632810

BankIt2503238 sts100 OK632811

BankIt2503238 sts101 OK632812

BankIt2503238 sts102 OK632813

BankIt2503238 sts103 OK632814

BankIt2503238 sts104 OK632815

BankIt2503238 sts105 OK632816

BankIt2503238 sts106 OK632817

BankIt2503238 sts107 OK632818

BankIt2503238 sts108 OK632819

BankIt2503238 sts109 OK632820

BankIt2503238 sts110 OK632821

BankIt2503238 sts111 OK632822

BankIt2503238 sts112 OK632823

BankIt2503238 sts113 OK632824

BankIt2503238 sts114 OK632825

BankIt2503238 sts115 OK632826

BankIt2503238 sts116 OK632827

BankIt2503238 sts117 OK632828

BankIt2503238 sts118 OK632829

BankIt2503238 sts119 OK632830

BankIt2503238 sts120 OK632831

BankIt2503238 sts121 OK632832

BankIt2503238 sts122 OK632833

BankIt2503238 sts123 OK632834

BankIt2503238 sts124 OK632835

BankIt2503238 sts125 OK632836

BankIt2503238 sts126 OK632837

BankIt2503238 sts127 OK632838

BankIt2503238 sts128 OK632839

BankIt2503238 sts129 OK632840

BankIt2503238 sts130 OK632841

BankIt2503238 sts131 OK632842

BankIt2503238 sts132 OK632843

BankIt2503238 sts133 OK632844

BankIt2503238 sts134 OK632845

BankIt2503238 sts135 OK632846

BankIt2503238 sts136 OK632847

BankIt2503238 sts137 OK632848

BankIt2503238 sts138 OK632849

BankIt2503238 sts139 OK632850

BankIt2503238 sts140 OK632851

BankIt2503238 sts141 OK632852

BankIt2503238 sts142 OK632853

BankIt2503238 sts143 OK632854

BankIt2503238 sts144 OK632855

BankIt2503238 sts145 OK632856

BankIt2503238 sts146 OK632857

BankIt2503238 sts147 OK632858

BankIt2503238 sts148 OK632859

BankIt2503238 sts149 OK632860

BankIt2503238 sts150 OK632861

BankIt2503238 sts151 OK632862

BankIt2503238 sts152 OK632863

BankIt2503238 sts153 OK632864

BankIt2503238 sts154 OK632865

BankIt2503238 sts155 OK632866

BankIt2503238 sts156 OK632867

BankIt2503238 sts157 OK632868

BankIt2503238 sts158 OK632869

BankIt2503238 sts159 OK632870

BankIt2503238 sts160 OK632871

BankIt2503238 sts161 OK632872

BankIt2503238 sts162 OK632873

BankIt2503238 sts163 OK632874

BankIt2503238 sts164 OK632875

BankIt2503238 sts165 OK632876

BankIt2503238 sts166 OK632877

BankIt2503238 sts167 OK632878

BankIt2503238 sts168 OK632879

BankIt2503238 sts169 OK632880

BankIt2503238 sts170 OK632881

BankIt2503238 sts171 OK632882

BankIt2503238 sts172 OK632883

BankIt2503238 sts173 OK632884

BankIt2503238 sts174 OK632885

BankIt2503238 sts175 OK632886

BankIt2503238 sts176 OK632887

BankIt2503238 sts177 OK632888

BankIt2503238 sts178 OK632889

BankIt2503238 sts179 OK632890

BankIt2503238 sts180 OK632891

BankIt2503238 sts181 OK632892

BankIt2503238 sts182 OK632893

BankIt2503238 sts183 OK632894

BankIt2503238 sts184 OK632895

BankIt2503238 sts185 OK632896

BankIt2503238 sts186 OK632897

BankIt2503238 sts187 OK632898

BankIt2503238 sts188 OK632899

BankIt2503238 sts189 OK632900

BankIt2503238 sts190 OK632901

BankIt2503238 sts191 OK632902

BankIt2503238 sts192 OK632903

BankIt2503238 sts193 OK632904

BankIt2503238 sts194 OK632905

BankIt2503238 sts195 OK632906

BankIt2503238 sts196 OK632907

BankIt2503238 sts197 OK632908

BankIt2503238 sts198 OK632909

BankIt2503238 sts199 OK632910

BankIt2503238 sts200 OK632911

BankIt2503238 sts201 OK632912

BankIt2503238 sts202 OK632913

BankIt2503238 sts203 OK632914

BankIt2503238 sts204 OK632915

BankIt2503238 sts205 OK632916

BankIt2503238 sts206 OK632917

BankIt2503238 sts207 OK632918

BankIt2503238 sts208 OK632919

BankIt2503238 sts209 OK632920

BankIt2503238 sts210 OK632921

BankIt2503238 sts211 OK632922

BankIt2503238 sts212 OK632923

BankIt2503238 sts213 OK632924

BankIt2503238 sts214 OK632925

BankIt2503238 sts215 OK632926

BankIt2503238 sts216 OK632927

BankIt2503238 sts217 OK632928

BankIt2503238 sts218 OK632929

BankIt2503238 sts219 OK632930

BankIt2503238 sts220 OK632931

BankIt2503238 sts221 OK632932

BankIt2503238 sts222 OK632933

BankIt2503238 sts223 OK632934

BankIt2503238 sts224 OK632935

BankIt2503238 sts225 OK632936

BankIt2503238 sts226 OK632937

BankIt2503238 sts227 OK632938

BankIt2503238 sts228 OK632939

BankIt2503238 sts229 OK632940

BankIt2503238 sts230 OK632941

BankIt2503238 sts231 OK632942

BankIt2503238 sts232 OK632943

BankIt2503238 sts233 OK632944

BankIt2503238 sts234 OK632945

BankIt2503238 sts235 OK632946

BankIt2503238 sts236 OK632947

BankIt2503238 sts237 OK632948

BankIt2503238 sts238 OK632949

BankIt2503238 sts239 OK632950

BankIt2503238 sts240 OK632951

BankIt2503238 sts241 OK632952

BankIt2503238 sts242 OK632953

BankIt2503238 sts243 OK632954

BankIt2503238 sts244 OK632955

BankIt2503238 sts245 OK632956

BankIt2503238 sts246 OK632957

BankIt2503238 sts247 OK632958

BankIt2503238 sts248 OK632959

BankIt2503238 sts249 OK632960

BankIt2503238 sts250 OK632961

BankIt2503238 sts251 OK632962

BankIt2503238 sts252 OK632963

BankIt2503238 sts253 OK632964

BankIt2503238 sts254 OK632965

BankIt2503238 sts255 OK632966

BankIt2503238 sts256 OK632967

BankIt2503238 sts257 OK632968

BankIt2503238 sts258 OK632969

BankIt2503238 sts259 OK632970

BankIt2503238 sts260 OK632971

BankIt2503238 sts261 OK632972

BankIt2503238 sts262 OK632973

BankIt2503238 sts263 OK632974

BankIt2503238 sts264 OK632975

BankIt2503238 sts265 OK632976

BankIt2503238 sts266 OK632977

BankIt2503238 sts267 OK632978

BankIt2503238 sts268 OK632979

BankIt2503238 sts269 OK632980

BankIt2503238 sts270 OK632981

BankIt2503238 sts271 OK632982

BankIt2503238 sts272 OK632983

BankIt2503238 sts273 OK632984

BankIt2503238 sts274 OK632985

BankIt2503238 sts275 OK632986

BankIt2503238 sts276 OK632987

BankIt2503238 sts277 OK632988

BankIt2503238 sts278 OK632989

BankIt2503238 sts279 OK632990

BankIt2503238 sts280 OK632991

BankIt2503238 sts281 OK632992

BankIt2503238 sts282 OK632993

BankIt2503238 sts283 OK632994

BankIt2503238 sts284 OK632995

BankIt2503238 sts285 OK632996

BankIt2503238 sts286 OK632997

BankIt2503238 sts287 OK632998

BankIt2503238 sts288 OK632999

BankIt2503238 sts289 OK633000

BankIt2503238 sts290 OK633001

BankIt2503238 sts291 OK633002

BankIt2503238 sts292 OK633003

BankIt2503238 sts293 OK633004

BankIt2503238 sts294 OK633005

BankIt2503238 sts295 OK633006

BankIt2503238 sts296 OK633007

BankIt2503238 sts297 OK633008

BankIt2503238 sts298 OK633009

BankIt2503238 sts299 OK633010

BankIt2503238 sts300 OK633011

BankIt2503238 sts301 OK633012

BankIt2503238 sts302 OK633013

BankIt2503238 sts303 OK633014

BankIt2503238 sts304 OK633015

BankIt2503238 sts305 OK633016

BankIt2503238 sts306 OK633017

BankIt2503238 sts307 OK633018

BankIt2503238 sts308 OK633019

BankIt2503238 sts309 OK633020

BankIt2503238 sts310 OK633021

BankIt2503238 sts311 OK633022

BankIt2503238 sts312 OK633023

BankIt2503238 sts313 OK633024

BankIt2503238 sts314 OK633025

BankIt2503238 sts315 OK633026

BankIt2503238 sts316 OK633027

BankIt2503238 sts317 OK633028

BankIt2503238 sts318 OK633029

BankIt2503238 sts319 OK633030

BankIt2503238 sts320 OK633031

BankIt2503238 sts321 OK633032

BankIt2503238 sts322 OK633033

BankIt2503238 sts323 OK633034

BankIt2503238 sts324 OK633035

BankIt2503238 sts325 OK633036

BankIt2503238 sts326 OK633037

BankIt2503238 sts327 OK633038

BankIt2503238 sts328 OK633039

BankIt2503238 sts329 OK633040

BankIt2503238 sts330 OK633041

BankIt2503238 sts331 OK633042

BankIt2503238 sts332 OK633043

BankIt2503238 sts333 OK633044

BankIt2503238 sts334 OK633045

BankIt2503238 sts335 OK633046

BankIt2503238 sts336 OK633047

BankIt2503238 sts337 OK633048

BankIt2503238 sts338 OK633049

BankIt2503238 sts339 OK633050

BankIt2503238 sts340 OK633051

BankIt2503238 sts341 OK633052

BankIt2503238 sts342 OK633053

BankIt2503238 sts343 OK633054

BankIt2503238 sts344 OK633055

BankIt2503238 sts345 OK633056

BankIt2503238 sts346 OK633057

BankIt2503238 sts347 OK633058

BankIt2503238 sts348 OK633059

BankIt2503238 sts349 OK633060

BankIt2503238 sts350 OK633061

BankIt2503238 sts351 OK633062

BankIt2503238 sts352 OK633063

BankIt2503238 sts353 OK633064

BankIt2503238 sts354 OK633065

BankIt2503238 sts355 OK633066

BankIt2503238 sts356 OK633067

BankIt2503238 sts357 OK633068

BankIt2503238 sts358 OK633069

BankIt2503238 sts359 OK633070

BankIt2503238 sts360 OK633071

BankIt2503238 sts361 OK633072

BankIt2503238 sts362 OK633073

BankIt2503238 sts363 OK633074

BankIt2503238 sts364 OK633075

BankIt2503238 sts365 OK633076

BankIt2503238 sts366 OK633077

BankIt2503238 sts367 OK633078

BankIt2503238 sts368 OK633079

BankIt2503238 sts369 OK633080

BankIt2503238 sts370 OK633081

BankIt2503238 sts371 OK633082

BankIt2503238 sts372 OK633083

BankIt2503238 sts373 OK633084

BankIt2503238 sts374 OK633085

BankIt2503238 sts375 OK633086

BankIt2503238 sts376 OK633087

BankIt2503238 sts377 OK633088

BankIt2503238 sts378 OK633089

BankIt2503238 sts379 OK633090

BankIt2503238 sts380 OK633091

BankIt2503238 sts381 OK633092

BankIt2503238 sts382 OK633093

BankIt2503238 sts383 OK633094

BankIt2503238 sts384 OK633095

BankIt2503238 sts385 OK633096

BankIt2503238 sts386 OK633097

BankIt2503238 sts387 OK633098

BankIt2503238 sts388 OK633099

BankIt2503238 sts389 OK633100

BankIt2503238 sts390 OK633101

BankIt2503238 sts391 OK633102

BankIt2503238 sts392 OK633103

BankIt2503238 sts393 OK633104

BankIt2503238 sts394 OK633105

BankIt2503238 sts395 OK633106

BankIt2503238 sts396 OK633107

BankIt2503238 sts397 OK633108

BankIt2503238 sts398 OK633109

BankIt2503238 sts399 OK633110

BankIt2503238 sts400 OK633111

BankIt2503238 sts401 OK633112

BankIt2503238 sts402 OK633113

BankIt2503238 sts403 OK633114

BankIt2503238 sts404 OK633115

BankIt2503238 sts405 OK633116

BankIt2503238 sts406 OK633117

BankIt2503238 sts407 OK633118

BankIt2503238 sts408 OK633119

BankIt2503238 sts409 OK633120

BankIt2503238 sts410 OK633121

BankIt2503238 sts411 OK633122

BankIt2503238 sts412 OK633123

BankIt2503238 sts413 OK633124

BankIt2503238 sts414 OK633125

BankIt2503238 sts415 OK633126

BankIt2503238 sts416 OK633127

BankIt2503238 sts417 OK633128

BankIt2503238 sts418 OK633129

BankIt2503238 sts419 OK633130

BankIt2503238 sts420 OK633131

BankIt2503238 sts421 OK633132

BankIt2503238 sts422 OK633133

BankIt2503238 sts423 OK633134

BankIt2503238 sts424 OK633135

BankIt2503238 sts425 OK633136

BankIt2503238 sts426 OK633137

BankIt2503238 sts427 OK633138

BankIt2503238 sts428 OK633139

BankIt2503238 sts429 OK633140

BankIt2503238 sts430 OK633141

BankIt2503238 sts431 OK633142

BankIt2503238 sts432 OK633143

BankIt2503238 sts433 OK633144

BankIt2503238 sts434 OK633145

BankIt2503238 sts435 OK633146

BankIt2503238 sts436 OK633147

BankIt2503238 sts437 OK633148

BankIt2503238 sts438 OK633149

BankIt2503238 sts439 OK633150

BankIt2503238 sts440 OK633151

BankIt2503238 sts441 OK633152

BankIt2503238 sts442 OK633153

BankIt2503238 sts443 OK633154

BankIt2503238 sts444 OK633155

BankIt2503238 sts445 OK633156

BankIt2503238 sts446 OK633157

BankIt2503238 sts447 OK633158

BankIt2503238 sts448 OK633159

BankIt2503238 sts449 OK633160

BankIt2503238 sts450 OK633161

BankIt2503238 sts451 OK633162

BankIt2503238 sts452 OK633163

BankIt2503238 sts453 OK633164

BankIt2503238 sts454 OK633165

BankIt2503238 sts455 OK633166

BankIt2503238 sts456 OK633167

BankIt2503238 sts457 OK633168

BankIt2503238 sts458 OK633169

BankIt2503238 sts459 OK633170

BankIt2503238 sts460 OK633171

BankIt2503238 sts461 OK633172

BankIt2503238 sts462 OK633173

BankIt2503238 sts463 OK633174

BankIt2503238 sts464 OK633175

BankIt2503238 sts465 OK633176

BankIt2503238 sts466 OK633177

BankIt2503238 sts467 OK633178

BankIt2503238 sts468 OK633179

BankIt2503238 sts469 OK633180

BankIt2503238 sts470 OK633181

BankIt2503238 sts471 OK633182

BankIt2503238 sts472 OK633183

BankIt2503238 sts473 OK633184

BankIt2503238 sts474 OK633185

BankIt2503238 sts475 OK633186

BankIt2503238 sts476 OK633187

BankIt2503238 sts477 OK633188

BankIt2503238 sts478 OK633189

BankIt2503238 sts479 OK633190

BankIt2503238 sts480 OK633191

BankIt2503238 sts481 OK633192

BankIt2503238 sts482 OK633193

BankIt2503238 sts483 OK633194

BankIt2503238 sts484 OK633195

BankIt2503238 sts485 OK633196

BankIt2503238 sts486 OK633197

BankIt2503238 sts487 OK633198

BankIt2503238 sts488 OK633199

BankIt2503238 sts489 OK633200

BankIt2503238 sts490 OK633201

BankIt2503238 sts491 OK633202

BankIt2503238 sts492 OK633203

BankIt2503238 sts493 OK633204

BankIt2503238 sts494 OK633205

BankIt2503238 sts495 OK633206

BankIt2503238 sts496 OK633207

BankIt2503238 sts497 OK633208

BankIt2503238 sts498 OK633209

BankIt2503238 sts499 OK633210

BankIt2503238 sts500 OK633211

BankIt2503238 sts501 OK633212

BankIt2503238 sts502 OK633213

BankIt2503238 sts503 OK633214

BankIt2503238 sts504 OK633215

BankIt2503238 sts505 OK633216

BankIt2503238 sts506 OK633217

BankIt2503238 sts507 OK633218

BankIt2503238 sts508 OK633219

BankIt2503238 sts509 OK633220

BankIt2503238 sts510 OK633221

BankIt2503238 sts511 OK633222

BankIt2503238 sts512 OK633223

BankIt2503238 sts513 OK633224

BankIt2503238 sts514 OK633225

BankIt2503238 sts515 OK633226

BankIt2503238 sts516 OK633227

BankIt2503238 sts517 OK633228

BankIt2503238 sts518 OK633229

BankIt2503238 sts519 OK633230

BankIt2503238 sts520 OK633231

BankIt2503238 sts521 OK633232

BankIt2503238 sts522 OK633233

BankIt2503238 sts523 OK633234

BankIt2503238 sts524 OK633235

BankIt2503238 sts525 OK633236

BankIt2503238 sts526 OK633237

BankIt2503238 sts527 OK633238

BankIt2503238 sts528 OK633239

BankIt2503238 sts529 OK633240

BankIt2503238 sts530 OK633241
